# A Bibliometric Analysis of Global Research on Japanese Encephalitis From 1934 to 2020

**DOI:** 10.3389/fcimb.2022.833701

**Published:** 2022-01-27

**Authors:** Chongxiao Xu, Weijia Zhang, Yuefeng Pan, Guowei Wang, Qikai Yin, Shihong Fu, Fan Li, Ying He, Songtao Xu, Zhenhai Wang, Guodong Liang, Kai Nie, Huanyu Wang

**Affiliations:** ^1^Department of Arboviruses, NHC Key Laboratory of Biosafety, National Institute for Viral Disease Control and Prevention, State Key Laboratory for Infectious Disease Prevention and Control, Chinese Center for Disease Control and Prevention, Beijing, China; ^2^Saint John’s Preparatory School, Collegeville, MN, United States; ^3^School of Clinical Medicine, Ningxia Medical University, Yinchuan, China; ^4^Department of Neurology, General Hospital of Ningxia Medical University, Engineering Research Center for Diagnosis and Treatment of Ningxia Nervous System Diseases, Yinchuan, China; ^5^Chinese Center for Disease Control and Prevention Wuhan Institute of Virology, Chinese Academy of Sciences Joint Research Center for Emerging Infectious Diseases and Biosafety, Center for Biosafety Mega-Science, Chinese Academy of Sciences, Wuhan, China

**Keywords:** Japanese encephalitis, Japanese encephalitis virus, bibliometric, visualization, research trends

## Abstract

Japanese encephalitis (JE) is a mosquito-borne disease caused by the Japanese encephalitis virus (JEV). The disease is mainly an epidemic in Asia and has been studied for nearly 90 years. To evaluate the research trends of JE, 3,023 English publications between 1934 and 2020 were retrieved and analyzed from the Web of Science database using indicators for publication, country or territory, citation, journal, author and affiliation, keyword co-occurrence cluster, and strongest citation bursts detection. The results of the bibliometric analysis and the visualization tools show that the number of annual publications on JE has been increasing. JE has been continuously studied in the USA and also many Asian countries, such as Japan, China, India, and South Korea; however, only a few publications have high citations. The main research groups of JE in the last 5 years were in China, Japan, and the UK. The keyword co-occurrence analysis and the strongest citation bursts detection revealed that most studies focused on the pathogenic mechanism of JEV, control of outbreaks, and immunization with JE vaccine. The research maps on JE obtained by our analysis are expected to help researchers effectively explore the disease.

## Introduction

Japanese encephalitis (JE) is an acute central nervous system infection caused by the Japanese encephalitis virus (JEV) and transmitted by mosquitoes. Patients with JE usually present with general symptoms, namely, fever, headache, and convulsions. Approximately 30% of patients with JE admitted to the hospital die, and around half of the survivors develop severe neurological sequelae, such as frank motor deficits, severe cognitive and language impairment, and further convulsions. In addition, Guillain-Barré syndrome has been reported to be associated with JEV infection in older age groups (Wang et al., 2020). JE is mainly prevalent in 24 countries in Southeast Asia and the Western Pacific Region. There are approximately 68,000 clinical cases each year, and more than 3 billion people are at risk of infection, which places a serious burden on the health of the population and healthcare economy (Campbell et al., 2011; Heffelfinger et al., 2017; Wu et al., 2021). JEV belongs to the *Flavivirus* genus of the Flaviviridae family and was first isolated from the brain of a fatal case of JE in 1935 (Solomon et al., 2000). The viral genome is a positive-sense, single-stranded RNA, which is approximately 11 kb in size. The genome comprises a single open reading frame (ORF) encoding a polyprotein that is processed into three structural proteins [capsid (C), precursor membrane or membrane (preM/M), and envelope (E)] and seven non-structural proteins (NS1, NS2a, NS2b, NS3, NS4a, NS4b, and NS5) (Sumiyoshi et al., 1987; Chambers et al., 1990). JEV can be divided into five genotypes (GI–GV) based on the E gene or the complete genome phylogenetic analysis (Solomon et al., 2003).

Bibliometrics is a cross-science that integrates mathematics, statistics, and philology, and analyzes all knowledge carriers quantitatively. The measuring parameters are mainly the amount of literature, authors, and words. Bibliometrics plays an important role in assisting relevant personnel in sorting out research trajectories, digging out the frontiers of disciplines, and finding research hotspots. It has been widely used in infectious disease research; however, JE has not been extensively reported until now.

The population composition of JE has changed from mainly children to several cases in adults across Japan, South Korea, China, and parts of India, as JE evolved into a vaccine-preventable disease (Campbell et al., 2011; Heffelfinger et al., 2017; Chen et al., 2021; Wu et al., 2021). Furthermore, the epidemic area of JE has expanded in recent years. Partial nucleic acid of JEV was detected in *Culex pipiens* pallens and birds in northeastern Italy in 2010, and in 2016, genotype III JEV nucleic acid was detected in the serum of patients in Angola, Africa, which had been earlier considered a non-endemic area for JE (Platonov et al., 2012; Ravanini et al., 2012; Simon-Loriere et al., 2017). The main epidemic genotype of JE is also changing. In Southeast Asia and India, genotype I has replaced genotype III as the main epidemic type of JEV (Gao et al., 2013; Schuh et al., 2013; Su et al., 2014). It is also noteworthy that the genotype V JEV was first isolated from the brain tissue of a patient in Malaysia who died from the disease in 1952, and was again isolated 57 years later from *Culex tritaeniorhynchus* mosquitoes in Nyingchi, Tibet, China. In 2011, the sequence of genotype V JEV was detected from *Culex bitaeniorhynchus* mosquitoes in South Korea, and finally, a case of GV JEV infection was found in South Korea in 2015 (Takhampunya et al., 2011; Li et al., 2011; Kim et al., 2015).

Aiming at the changing epidemiological characteristics of viruses and diseases, this article summarizes the studies related to JE from the aspects of publication, country or territory, citation, journal, author and affiliation, keyword co-occurrence cluster, keywords with the strongest citation bursts, etc., using bibliometric and visualization methods. The study seeks to sort out the research process and frontiers, and thereby serve as a valuable reference and guidance for researchers in the future.

## Materials and Methods

### Sources of Literature Data

The data of this study was derived from the Science Citation Index Expanded (SCI-Expanded) database of the Web of Science (WoS), USA Institute of Scientific Information. It contains all the documents indexed by SCI-EXPANDED since 1900. The literature data search was conducted on August 11, 2021.

### Search Strategy

According to the search rules of WoS, the search condition was set to (“JAPANESE ENCEPHALITIS” OR “JAPANESE B ENCEPHALITIS”) (Title) or (“JAPANESE ENCEPHALITIS” OR “JAPANESE B ENCEPHALITIS”) (Author Keywords) and Meeting Abstracts (Exclude Document Types) and English (Languages), and the retrieval time span was January 1, 1900 to December 31, 2020. After the retrieval, some pieces of literature with poor relevance to the research topic were removed through manual screening.

### Data Analysis

All the essential literature data and information for this research were fully recorded and exported in txt. and xlsx. format on August 11, 2021. Microsoft Excel (version 16.46) was used to perform basic descriptive analysis (such as the number of publications per year, publications with the most citations, etc.). VOSviewer (version 1.6.15) was used to generate visual maps. In the author analysis, researchers with a number of publications greater than 8, and the strength of association with other authors greater than 20 were included in the graph; before keyword analysis, synonyms were merged and invalid words were eliminated by constructing a synonym dictionary. CiteSpace (version 5.8.R3) was used to analyze the keywords with the strongest citation bursts and summarize the research hotspots and frontiers related to JE.

## Results

### Analysis of the Publication Numbers and Countries

**Figure 1:**
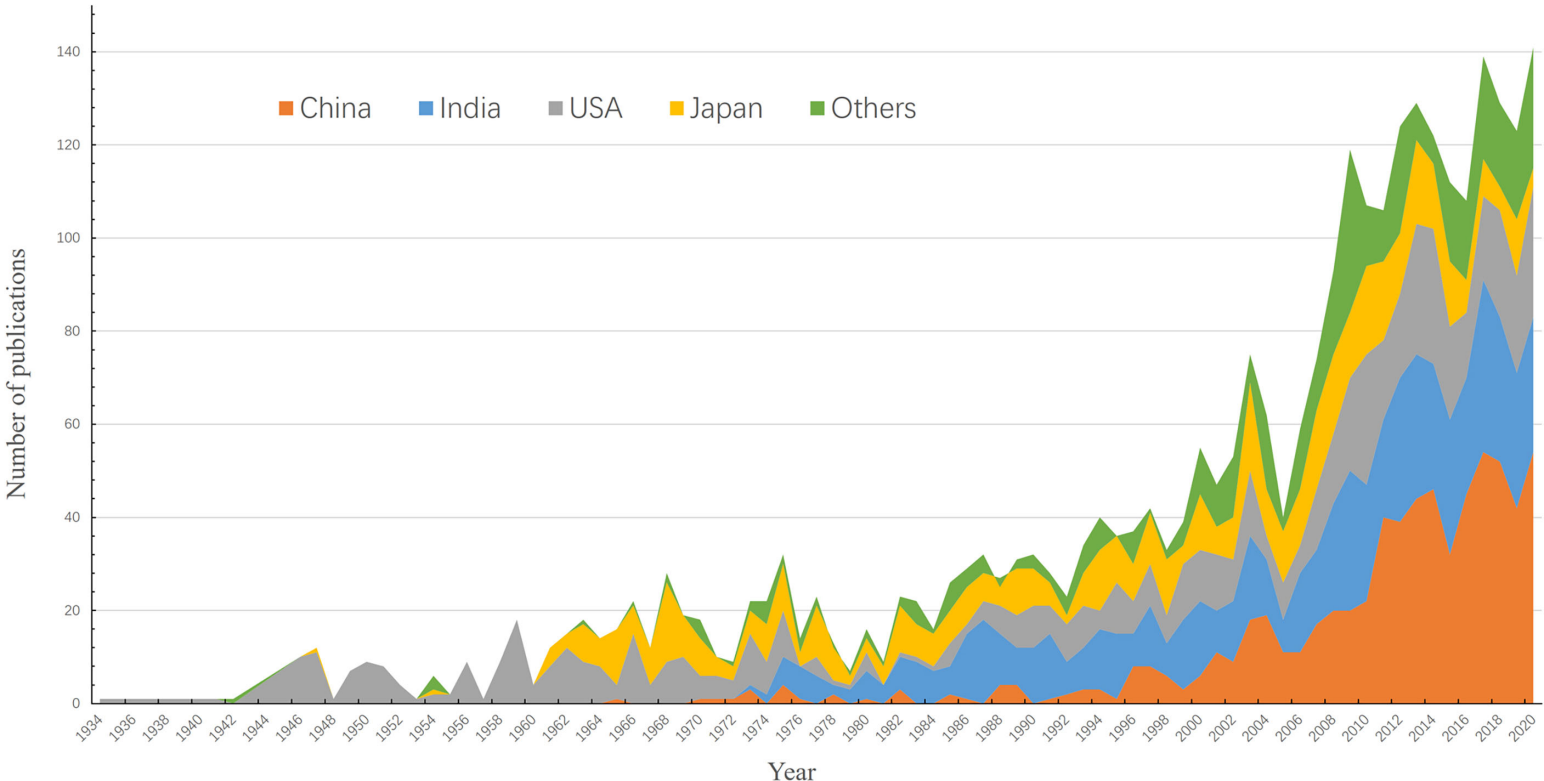
Number of literatures published each year. The abscissa represents the publication year, the ordinate represents the publication amount, and the colors are used to code the publications from different countries.

A total of 3,023 publications meeting the search requirements were obtained from 1934 to 2020. The annual number of publications has been increasing and the countries with the most publications are India (704), the USA (697), China (676), and Japan (543). Based on the annual number of publications, JE research can be roughly divided into three stages. The period of 1934–1960 was the initial stage of research, when the WoS first included an article named *St. Louis encephalitis* (Webster and Fite, 1934) that was published in *Science* in 1934. During this period, the number of publications remained low with the total being 108, and the average being approximately four per year. The United States was the most important country for JE research in this period. The 1960s to the end of the 20th century represented the developmental stage for JE research. The number of publications on JE was 898, and the average was approximately 23 per year. India, Japan, and China got involved in JE research during this stage. From 2000 to 2020, the publications on JE entered into a stage of rapid development. The total number of publications was 2,017, and the average was approximately 96 per year. In 2000, there were more than 50 new publications, and the number exceeded 100 in 2009. From 2009 to 2020, it remained above 100 and increased to 141 by 2020. Among them, publications in China accounted for 30.34% (612/2,017), making it the most important country for JE research over the past 20 years (**Figure 1**).

### Analysis of Citations

The top 10 reviews with the most citations from 1934 to 2020 were cited 4,082 times, accounting for more than 52% (4,082/7,783) of relevant reviews. The most cited review, *Emerging Flaviviruses: The Spread and Resurgence of Japanese Encephalitis, West Nile and Dengue Viruses* (Mackenzie et al., 2004), received 952 total citations and 59.5 average citations per year. There were only 3 reviews with more than 500 citations, 2 reviews with 300–500 citations, and 5 reviews with 200–300 citations. The latest publication among the 10 most frequently cited reviews was published in 2011 (**Table 1**).

**Table 1:** Top 10 reviews with the most citations.

Rank^1^	Author^2^	Country	Article Title	Source Title	Publication Year	Total Citations	AC/EY^3^
1	Mackenzie et al.	Australia	Emerging Flaviviruses: The Spread and Resurgence of Japanese Encephalitis, West Nile and Dengue Viruses	Nature Medicine	2004	952	59.5
2	Gubler et al.	USA	The Global Emergence/Resurgence of Arboviral Diseases as Public Health Problems	Archives of Medical Research	2002	668	37.11
3	Campbell et al.	USA	Estimated Global Incidence of Japanese Encephalitis: A Systematic Review	Bulletin of the World Health Organization	2011	509	56.56
4	Erlanger et al.	Switzerland	Past, Present, and Future of Japanese Encephalitis	Emerging Infectious Diseases	2009	365	33.18
5	Solomon et al.	England	Japanese Encephalitis	Journal of Neurology Neurosurgery and Psychiatry	2000	352	17.6
6	van den Hurk et al.	Australia	Ecology and Geographical Expansion of Japanese Encephalitis Virus	Annual Review of Entomology	2009	295	26.82
7	Vaughn and Hoke	USA	The Epidemiology of Japanese Encephalitis-Prospect for Prevention	Epidemiologic Reviews	1992	284	10.14
8	Misra et al.	India	Overview: Japanese Encephalitis	Progress in Neurobiology	2010	230	23
9	Hombach et al.	Switzerland	Report on a WHO Consultation on Immunological Endpoints for Evaluation of New Japanese Encephalitis Vaccines, WHO, Geneva, 2-3 September, 2004	Vaccine	2005	224	14.93
10	Guy et al.	France	Preclinical and Clinical Development of YFV 17D-Based Chimeric Vaccines Against Dengue, West Nile and Japanese Encephalitis Viruses	Vaccine	2010	203	20.3

^1^Ranked by total citations.

^2^Names of the corresponding authors (on Web of Science) were provided. If there are more than one corresponding author, the last one is shown.

^3^Average citations per year.

The top 15 articles with the most citations from 2005 to 2020 were cited a total of 2,205 times, accounting for more than 8% of relevant research articles (2,205/27,149). The most cited article, *Proinflammatory Mediators Released by Activated Microglia Induces Neuronal Death in Japanese Encephalitis* (Ghoshal et al., 2007), had 265 total citations and 20.38 average citations per year. Except for the first, the other articles were cited less than 200 times. According to the average number of citations per year, there were 2 articles that were cited more than 20 times. In addition, the latest article among the top 15 was published in 2015 with 109 citations, of which ten were published before 2010 (**Table 2**).

**Table 2:** Top 15 articles with the most citations from 2005 to 2020.

Rank^1^	Author^2^	Country	Article Title	SourceTitle	Publication Year	Total Citations	AC/EY^3^
1	Ghoshal et al.	India	Proinflammatory Mediators Released by Activated Microglia Induces Neuronal Death in Japanese Encephalitis	Glia	2007	265	20.38
2	Lin et al.	China	Blocking of Interferon-Induced Jak-Stat Signaling by Japanese Encephalitis Virus NS5 Through a Protein Tyrosine Phosphatase-Mediated Mechanism	Journal of Virology	2006	196	14
3	Lin et al.	China	Flavivirus Induces Interferon-Beta Gene expression Through a Pathway Involving RIG-I-dependent IRF-3 and PI3K-Dependent NF-kappa B Activation	Microbes and Infection	2006	186	13.29
4	Wang et al.	China	Molecular Epidemiological Analysis of Japanese Encephalitis Virus in China	Journal of General Virology	2007	167	12.85
5	Ghoshal et al.	India	Antiviral and Anti-Inflammatory Effects of Rosmarinic Acid in an Experimental Murine Model of Japanese Encephalitis	Antimicrobial Agents and Chemotherapy	2007	158	12.15
6	Luca et al.	USA	Crystal Structure of the Japanese Encephalitis Virus Envelope Protein	Journal of Virology	2012	144	18
7	Lu et al.	China	Crystal Structure of the Full-Length Japanese Encephalitis Virus NS5 Reveals a Conserved Methyltransferase-Polymerase Interface	Plos Pathogens	2013	135	19.29
8	Tauber et al.	Austria	Safety and Immunogenicity of a Vero-Cell-Derived, Inactivated Japanese Encephalitis vaccine: A Non-Inferiority, Phase III, Randomised Controlled Trial	Lancet	2007	130	10
8	Lee et al.	Austria	Antiviral Effect of the Heparan Sulfate Mimetic, PI-88, Against Dengue and Encephalitic Flaviviruses	Antiviral Research	2006	130	9.29
10	Melian et al.	Austria	NS1’ of Flaviviruses in the Japanese Encephalitis Virus Serogroup Is a Product of Ribosomal Frameshifting and Plays a Role in Viral Neuroinvasiveness	Journal of Virology	2010	129	12.9
11	Pan et al.	China	Emergence of Genotype I of Japanese Encephalitis Virus as the Dominant Genotype in Asia	Journal of Virology	2011	123	13.67
12	Parida et al.	India	Japanese Encephalitis Outbreak, India, 2005	Emerging Infectious Diseases	2006	113	8.07
13	Ghoshal et al.	India	Minocycline Neuroprotects, Reduces Microglial Activation, Inhibits Caspase 3 Induction, and Viral Replication Following Japanese Encephalitis	Journal of Neurochemistry	2008	111	9.25
14	Li et al.	China	Viral Infection of the Central Nervous System and Neuroinflammation Precede Blood–Brain Barrier Disruption during Japanese Encephalitis Virus Infection	Journal of Virology	2015	109	21.8
14	Firth and Atkins	USA	A Conserved Predicted Pseudoknot in the NS2A-Encoding Sequence of West Nile and Japanese Encephalitis Flaviviruses Suggests NS1 ‘ May Derive from Ribosomal Frameshifting	Virology Journal	2009	109	9.91

^1^Ranked by total citations.

^2^Names of the corresponding authors (on Web of Science) were provided. If there are more than one corresponding author, the last one is shown.

^3^Average citations per year.

### Analysis of Journals

All publications included in the study came from 561 different journals and the top 10 journals with the most publications were listed. These 10 journals produced 29.34% (887/3,023) of all publications analyzed in this study and 37.29% (27,172/72,869) of the total citations. Among them, the journal *Vaccine* has published the maximum number of papers related to JE (165) and the *Journal of Virology* has the highest average citation frequency (65.19) (**Table 3**).

**Table 3:** Top 10 journals with the most publications.

Rank^1^	Journal	Publication numbers	Total citations^2^	Citations per publication^3^	H-index of publications^4^
1	Vaccine	165	4,352	26.38	38
2	American Journal of Tropical Medicine and Hygiene	138	4,944	35.83	38
3	Indian Journal of Medical Research	106	1,376	12.98	20
4	Journal of Virology	98	6,389	65.19	52
5	Acta Virologica	79	703	8.9	14
6	Journal of General Virology	77	2,970	38.57	34
7	Virology	71	3,862	54.39	35
8	Plos One	55	999	18.16	19
9	Archives of Virology	51	731	14.33	15
10	Virus Research	47	846	18	19

^1^Ranked by the publication number.

^2^Total citations mean the sum of citations received these years (before our research time) about those publications (the publication numbers column in this table) in each journal.

^3^Citations per publication was calculated according to the publication numbers and their total citations.

^4^H-index of Publications means that there are “h” publications that have been cited at least “h” time.

### Analysis of Authors and Affiliations

**Figure 2:**
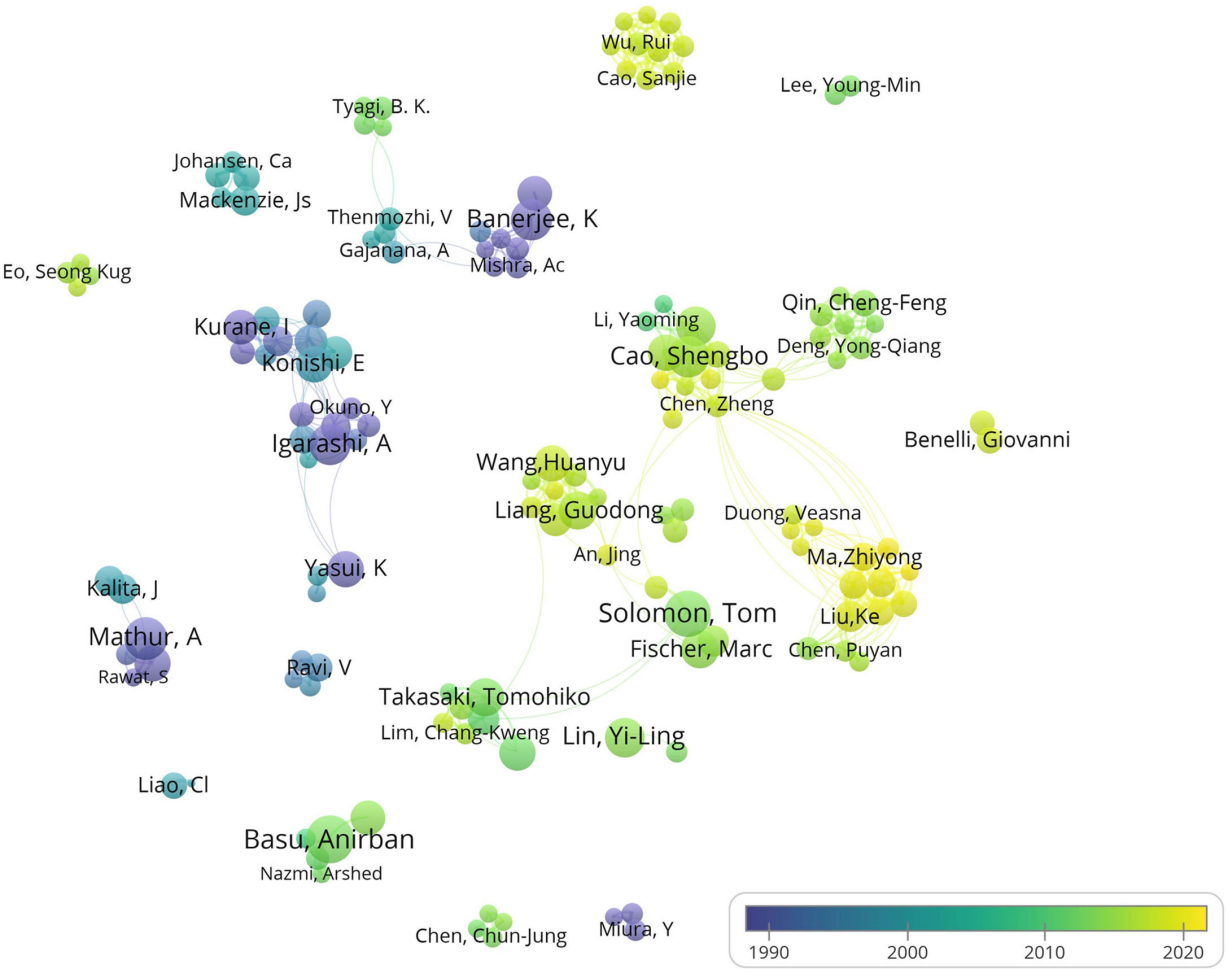
Author co-authorship analysis map. The size of each node indicates the number of publications produced by the author. The thickness of each link indicates the strength of collaboration relationship between two authors. The distance between two nodes indicates the relatedness of the links they each has. Indicator shows the publication time of the author from purple to yellow.

The groups led by the Japanese scientists Konishi E. (Kyoto Prefectural University of Medicine), Kurane I. (National Institute of Infectious Diseases), and Igarashi A. (Showa University), and the Indian scientists Mathur A. (Saraswati Dent & Med Coll) and Banerjee K. (ICAR—National Research Centre for Grapes), were the most productive research authors in the field of JE in the period of 1990 to 2000. From 2000 to 2010, groups led by scientists, such as Solomon T. from the University of Liverpool, Basu A. from the National Brain Research Center of India, and Tomohiko T. from the National Institute of Infectious Diseases in Japan, were the most active JE researchers. Since 2010, Chinese scientists, such as GD Liang and HY Wang from the Chinese Center for Disease Control and Prevention, SB Cao from Huazhong Agricultural University, and CF Qin from the Beijing Institute of Microbiology and Epidemiology, have become the most active researchers in the field of JE. In addition, the team of ZY Ma from the Chinese Academy of Agricultural Sciences joined JE research around 2020. The top 15 institutions with the most publications are listed in **Table 4**, with most of them were from the United States, India, China, Japan, and Thailand (**Figure 2**, **Table 4**).

**Table 4:** Top 15 Affiliations with the most publications.

Rank^1^	Affiliation	Country/Territory	Publication numbers	Total citations^2^	Citations per publication^3^	Distribution of years^4^		
						1961–1990	1991–2005	2006–2020
1	ICMR-National Institute of Virology (NIV)	India	170	2,247	13.22	84	45	41
2	Indian Council of Medical Research (ICMR)	India	111	1,738	15.66	2	36	73
3	United States Department of Defense	USA	103	6,048	58.72	35	39	29
4	Department Of Biotechnology (DBT) India	India	102	3,862	37.86	0	14	88
5	Walter Reed Army Institute of Research (WRAIR)	USA	94	5,676	60.38	31	35	28
6	Centers for Disease Control and Prevention-USA	USA	89	4,830	54.27	0	23	66
7	United States Army	USA	88	5,762	65.48	29	35	24
8	National Institute of Infectious Diseases (NIID)	Japan	86	2,485	28.9	0	25	61
9	Mahidol University	Thailand	81	2,114	26.1	6	9	66
10	Huazhong Agricultural University	China	68	1,424	20.94	0	3	65
11	Nagasaki University	Japan	65	1,958	30.12	12	20	33
12	Armed Forces Research Institute Of Medical Science (AFRIMS)	Thailand	64	3,944	61.63	23	18	23
13	Chinese Center for Disease Control and Prevention	China	62	1,950	30.95	1	1	60
13	National Defense Medical Center	Taiwan (China)	62	3,302	53.26	0	37	25
15	Sanjay Gandhi Postgraduate Institute of Medical Sciences	India	59	1,666	28.24	0	25	34

^1^Ranked by publication numbers.

^2^Total citations mean the sum of citations received these years (before our research time) about the publications (the publication numbers column in this table) of each author.

^3^Citations per publication was calculated according to the publication numbers and their total citations.

^4^Distribution of years means the publication numbers published in 1961–1990, 1991–2005, and 2006–2020 respectively.

### Analysis of Keywords

**Figure 3:**
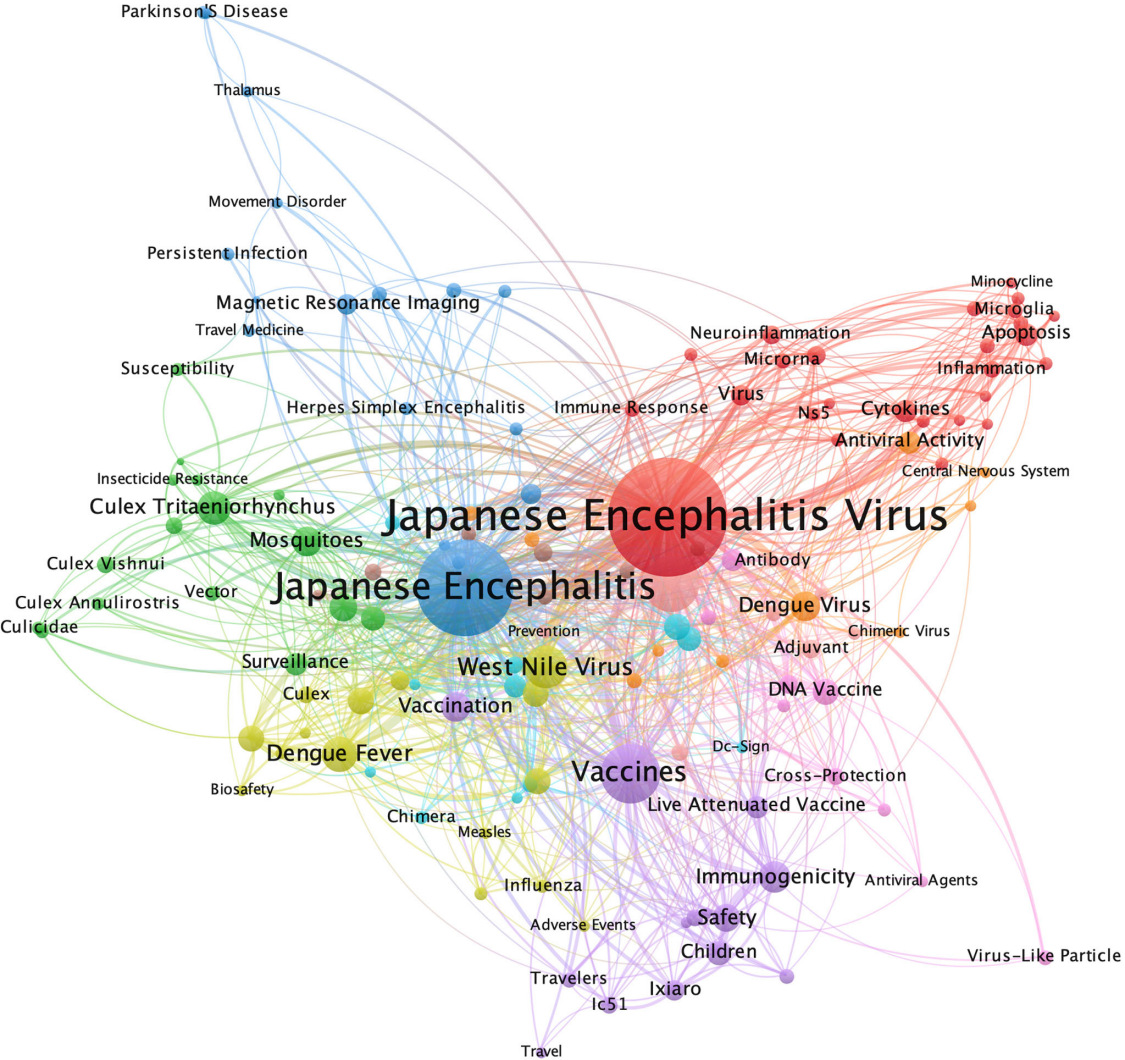
Keyword co-occurrence analysis map. The size of each node indicates the occurrence of the keyword in all 3,023 publications. The thickness of each link indicates the strength of co-occurrence relationship between two keywords. The distance between two nodes indicates the relatedness of the links they each has. Nodes with common attributes are assigned to a color-coded cluster.

The research directions of JE-related publications collected by WoS were divided into ten clusters, four of which were dominant. The keywords which roughly connected to JEV were shown in red, and the word “Japanese encephalitis virus” occurred 737 times, accounting for 24.38% (737/3,023) of the total publications. The keywords which roughly connected to JE were shown in blue and “Japanese encephalitis” appeared 470 times, accounting for 15.55% (470/3,023) of the total publications. “Vaccines” appeared 164 times, accounting for 5.43% (164/3,023) of the total publications and the words relevant to vaccines and immunization were coded with purple. The keywords related to the pathogen vectors were shown in green and “*Culex* mosquitoes” appeared 87 times, accounting for 2.88% (87/3,023) of the total publications (**Figure 3**).

**Figure 4:**
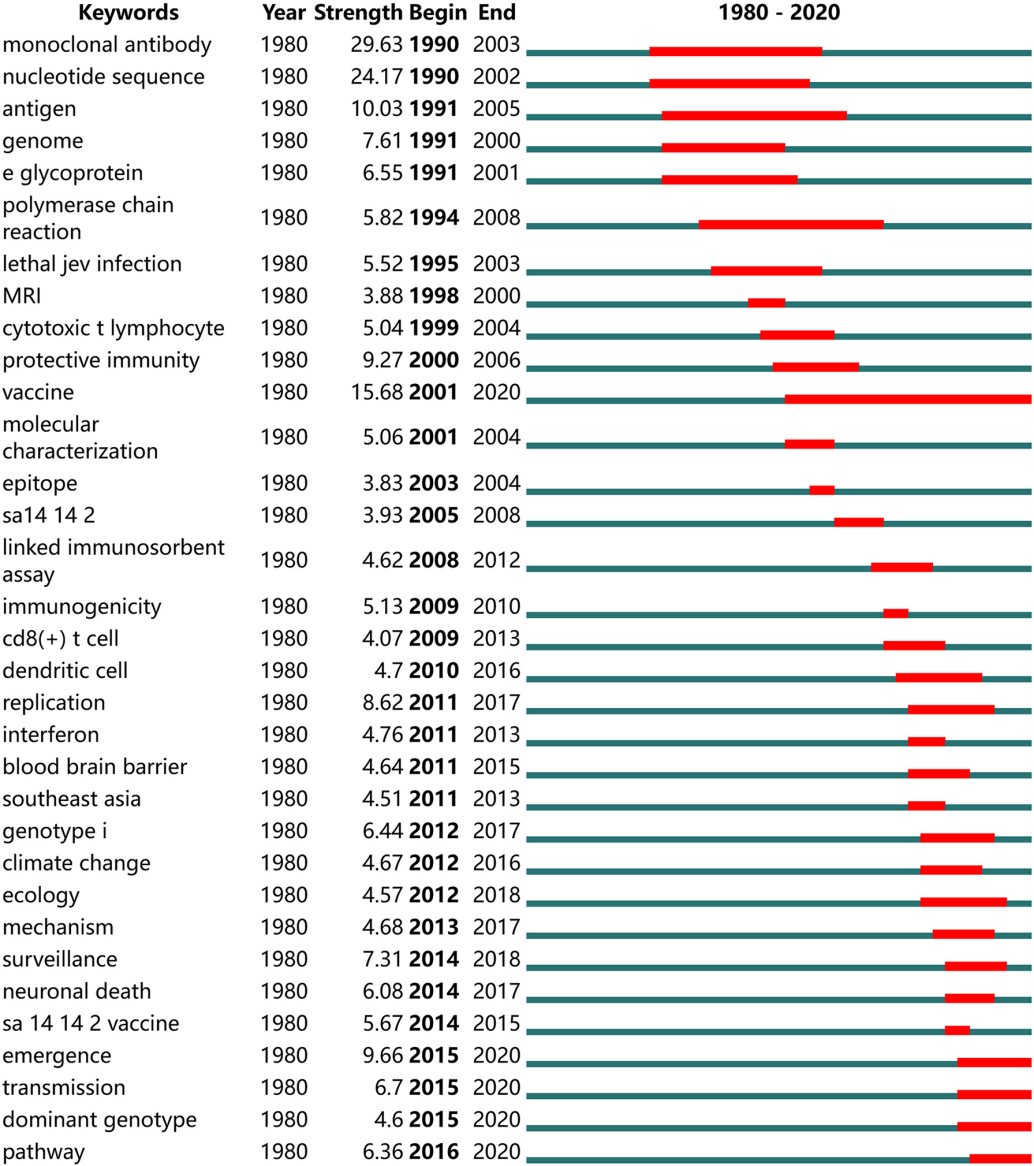
Keywords with the strongest citation burst in publications on JE research from 1980 to 2020. The time interval is depicted as a blue line. The period in which a keyword is found to exhibit a strong increase is shown as a red line, which indicates the beginning and the end year of the burst.

A total of 33 keywords with the strongest citation bursts were detected. There were 10 keywords (“monoclonal antibody”, “antigen”, “e glycoprotein”, “cytotoxic t lymphocyte”, “protective immunity”, “vaccine”, “epitope”, “immunogenicity”, “cd8 (+) t cell”, “sa 14-14-2 vaccine”) related to the topic of immunization and vaccine development were detected, accounting for 30.3% (10/33). “Monoclonal antibody” was the strongest keyword, followed by “nucleotide sequence”, “antigen” and “vaccine”, each of which had an outbreak range of more than 10 years. “Emergence”, “transmission”, “dominant genotype” and “pathway” were the most recent burst keywords (**Figure 4**).

## Discussion

As a vaccine-preventable infectious disease, JE has a great impact on public health in Asia due to its high fatality rate, high disability rate, and potential global epidemic risk. There are still major challenges in rapid and effective disease control and prevention of JE once an epidemic occurs. Since the 1930s, the isolation of JEV had promoted research on JE, and a large volume of literature was published. In order to improve research efficiency and reduce unnecessary duplication, 3,023 publications that meet the research objectives were analyzed by innovative bibliometric and visual tools to uncover the research trends and hotspots, promote cooperation between JE researchers in different teams, and provide reference and guidance for future researchers.

The annual publication volumes reflect the key development of this field over the past years (Durieux and Gevenois, 2010). Between 1934 and 2020, the number of publications remained low, but it continued to grow. There was an explosive growth peak around 2009, and this may be related to the establishment of WHO reference laboratories for JE in 2009 in the Southeast Asian and Western Pacific Region, which promoted the monitoring and research of JE in relevant countries.

Asian countries are highly concerned about JE because of conditions, such as epidemic areas, distribution of vectors, climate, geographical location, social health, and economics. Meanwhile, the United States has continued to pay attention to JE and has carried out comprehensive and extensive research. Since the 1960s, many Asian countries such as China, India, and Japan have been involved in JE research, accompanied by their inherent economic and social development. In recent years, Asian countries have played a more important role in the research of JE. However, because this research is mainly aimed at the English publications of WoS, publications in other languages such as Japanese or Chinese were excluded, which has a certain impact on the accuracy of the analysis about different countries.

The number of citations of a publication reflects its dissemination and influence to a certain extent (Muniz et al., 2018). A large number of JE publications were lowly cited, whereas highly cited publications were published earlier. This shows that JE is not a global point of focus because of its somewhat limited epidemic area. There are only a few research groups engaged specifically in JE research worldwide, most of which are in the field of disease prevention and control, such as the Centers for Disease Control and Prevention in the USA and China, and the National Institute of Infectious Diseases in Japan. Furthermore, in-depth exploration of specialized fields may change the focus and direction of future research in this field. Among the 15 most cited articles listed in **Table 2**, there are studies on different topics including but not limited to antiviral drugs, vaccine development, molecular epidemiology, and virus structure; the pathogenic mechanism of the virus has become more popular since JE research has entered the stage of rapid development.

Journals with a high frequency of publication of related literature provide researchers with publication guidelines for documents (Deng et al., 2020). Among the 15 most cited articles listed in **Table 2**, five articles were from the *Journal of Virology*, which reflects the authority and high degree of attention to the field by the journal. In addition, the journal *Vaccine* has published the maximum number of papers related to JE, indicating that vaccine development and application is a popular topic in JE research.

The analysis of the authors helps to understand a research field more comprehensively and evaluate the contributions, research level, and the academic status of researchers in the field objectively (Podsakoff et al., 2008). Our results show that since 2010, the following JE research groups are the most representative: Solomon T. of the University of Liverpool in the UK is mainly engaged in research involving JE epidemiology (Ooi et al., 2008), pathogenesis and clinical features (Solomon and Vaughn, 2002), diagnosis and treatment methods (Solomon et al., 1998; Turtle and Solomon, 2018), immune mechanism (Turtle et al., 2016), transmission vector (Mackenzie-Impoinvil et al., 2015) and molecular epidemiology of JEV (Mohammed et al., 2011); Basu A. of the National Brain Research Center in India focuses on the pathogenesis of JEV (Mackenzie et al., 2004) and the pathology and clinical features (Ghosh and Basu, 2009) of JE; Takasaki T of the National Institute of Infectious Diseases in Japan is mainly devoted to the serological detection of the JEV antibody (Hamano et al., 2007), vaccine and immunization (Kurane and Takasaki, 2000), epidemiology survey (Kurane et al., 2013), etc. In addition, there are mainly the following groups in China: GD Liang and HY Wang of the Chinese Center for Disease Control and Prevention are mainly engaged in the analysis of the JEV genotype and molecular epidemiology (Wang et al., 2007; Pan et al., 2011; Gao et al., 2019), JE epidemiology, and disease prevention and control-related research (Yin et al., 2010; Gao et al., 2014; Wang and Liang, 2015). The team of SB Cao from the Huazhong Agricultural University is mainly engaged in basic research related to JEV pathogenic mechanism (Li et al., 2015), immune response (Cao et al., 2011), and antiviral drugs (Ye et al., 2014). CF Qin from the Beijing Institute of Microbiology and Epidemiology is mainly devoted to research on JEV infection and pathogenic mechanism, and relevant research on the JEV live attenuated vaccine SA14-14-2 (Ye et al., 2012; Li et al., 2013).

Keyword co-occurrence analysis and citation burst detection can provide a large amount of meaningful information to help researchers identify hotspots and trends in their fields (Hand et al., 2002; Romero and Portillo-Salido, 2019). Judging from the keyword clustering map and the citation burst detection, immunization, vaccine and vector of JEV are the fields that have attracted continuous attention in JE research, while emerging virus, JEV transmission, dominant genotypes, pathogenic mechanism and immune pathways can be the popular research trends in future. Recent years, the main epidemic genotype of JEV has gradually changed from genotypes III to I. The genotype V JEV has also received increasing attention because of the re-emergence of clinical cases of the genotype V JEV infection. The vaccines used across the world were developed based on genotype III JEV, which has a low protective efficacy against the genotype V (Li et al., 2014; Tajima et al., 2015; Cao et al., 2016), and the protective effect for genotype II or IV has not been evaluated. Therefore, research on multivalent vaccine for different genotypes of JEV may become the focus of future studies. Furthermore, with the widespread application of vaccines, the incidence of infectious cases in children significantly declined, and the growing number of adult cases has become a significant public health problem. Immunocompromised populations, organ transplantation and vaccine breakthrough cases are poised to attract attention in the JE research field (Cheng et al., 2018; Qi et al., 2020).

The current explosion of information and the unprecedented amount of data makes it challenging for researchers to derive actual benefit. Bibliometric analysis combined with data visualization is critical for exploring and communicating information effectively and for helping researchers to continue their progress (Wong et al., 2012). Therefore, we conducted a bibliometric and visual analysis of the publications about JE from 1934 to 2020, which might promote cooperation among researchers and provide valuable ideas for the future research of JE.

## Data Availability Statement

The original contributions presented in the study are included in the article/supplementary material. Further inquiries can be directed to the corresponding authors.

## Author Contributions

HW and KN conceived and designed the analysis. CX, WZ, YP, FL, and YH collected the data. CX, WZ, YP, and GW analyzed the data. CX, WZ, QY, and SF wrote the draft. CX, SX, ZW, GL, KN, and HW modified the draft. All authors contributed to the article and approved the submitted version.

## Funding

This research was funded by the Science and Technology Key Research Program of Ningxia, China (2019BCG01003 to ZW, HW), the Development Grant of State Key Laboratory of Infectious Disease Prevention and Control (2015SKLID505 to HW). The funders had no role in study design, data collection and analysis, decision to publish, or preparation of the manuscript.

## Conflict of Interest

The authors declare that the research was conducted in the absence of any commercial or financial relationships that could be construed as a potential conflict of interest.

## Publisher’s Note

All claims expressed in this article are solely those of the authors and do not necessarily represent those of their affiliated organizations, or those of the publisher, the editors and the reviewers. Any product that may be evaluated in this article, or claim that may be made by its manufacturer, is not guaranteed or endorsed by the publisher.
